# Design of Decanoic Acid/Polysorbate 80 Composite Vesicles as Cosmetics Carrier: Stability, Skin Permeability, Antioxidant and Antibacterial Activity

**DOI:** 10.3390/molecules30030624

**Published:** 2025-01-31

**Authors:** Ying Yang, Bohang Zou, Xinyu Fan, Xinyue Ma, Siqi Li, Xiangyu Zhang, Jinlian Li, Dongmei Wu

**Affiliations:** 1 College of Pharmacy, Jiamusi University, Jiamusi 154007, China; 18249574539@163.com (Y.Y.); zbh11302025@163.com (B.Z.); 13339481413@163.com (X.F.); 18249143626@163.com (X.M.); 15344549861@163.com (S.L.); lijinlian@jmsu.edu.cn (J.L.); dmwu@jmsu.edu.cn (D.W.); 2 Heilongjiang Provincial Key Laboratory of New Drug Development and Pharmacotoxicological Evaluation, Jiamusi University, Jiamusi 154007, China

**Keywords:** fatty acid vesicle, cosmetics carrier, skin permeability, antibacterial activity

## Abstract

Fatty acid vesicles are natural biomaterials which possess unique bilayer structures and offer biomimetic advantages for drug and gene delivery. Nevertheless, the formation of fatty acid vesicles is limited to neutral alkaline circumstances and cannot adapt to the acidic environment of the living system. In this work, the non-ionic surfactant polysorbate 80 (TW80) was introduced, extending the pH window of vesicles formed by decanoic acid (DA) from 6.90–7.80 to 2.28–6.31. The DA/TW80 composite vesicles were used to encapsulate quercetin (QT), achieving an encapsulation efficiency of up to 75.6%. The formation of DA/TW80/QT composite vesicles was confirmed through Fourier transform infrared spectroscopy, differential scanning calorimetry, and X-ray diffraction. Moreover, free QT was released rapidly, while QT encapsulated in the DA/TW80 composite vesicles demonstrated a slower release profile. Skin permeability studies revealed that the cumulative drug penetration within 24 h using the DA/TW80/QT composite vesicles reached approximately 904.7 μg·cm^−2^, 1.81 times higher than that of a QT solution. Furthermore, the DA/TW80/QT composite vesicles demonstrated enhanced antioxidant activity and greater antibacterial efficacy compared to either the drug or the vesicles alone. The results provide a crucial foundation for the application of drug-loaded vesicles in cosmetics.

## 1. Introduction

Functional molecular self-assembled technologies have attracted significant attention, providing the foundation for building well-defined structures on the nanometer length scale. Fatty acids constitute a significant component of lipids within the body and are abundantly present in diverse plant and animal species [1,2]. In 1973, Gebicki and Hicks initially prepared fatty acid vesicles (FAVs) using unsaturated fatty acid salts by mixing oleic acid and oleate in water [3]. Further investigations have demonstrated that saturated fatty acids can also form vesicles at an appropriate pH [4,5]. Therefore, FAVs can spontaneously assemble into closed double-layer structures in water by molecular self-assembly [6,7]. The use of FAVs has grown considerably in recent years due to its wide availability, high stability, skin permeability, and drug loading capacity. The simplicity of the FAVs fabrication process, excellent carboxylate stimulation response, hollow structure, and unique biocompatibility suggest that they have great potential for applications in food, drug delivery, cosmetics, and other industries [8,9,10,11].

In the study of FAV, decanoic acid (DA), as a medium-chain fatty acid, provides a hydrophobic environment that helps to form stable vesicle membranes and has good emulsifying properties, making it suitable for loading hydrophobic drugs. It has attracted attention due to its low toxicity and wide range of sources, and its potential application as a natural green reagent in cosmetics should not be underestimated [12]. However, the pH window of DA vesicles is formed only under neutral to alkaline conditions, which is not adapted to the acidic environment of living systems [4]. In addition, the pH window range is only one unit, which is prone to rupture with changes in pH [13,14]. Walde and Samsung [15] showed that the addition of an equimolar amount of sodium dodecyl benzene sulfonate (SDBS) to decanoic acid solution extended the pH range of vesicles to 4.3–7.5. Caschera et al. [16] discovered that the addition of dodecyltrimethylammonium bromide (DTAB) migrated the range of pH values at which vesicles formed to 5.4–8.6. This suggests that the addition of surfactants is able to migrate and expand the pH window for FAVs.

Recently, it has been discovered that FAVs can improve the bioavailability of active substances and functions for topical delivery. Quercetin (QT) is a natural flavonoid [17] with various biological activities and is widely used in cosmetics. Its main advantages include antioxidant, anti-inflammatory, anti-aging, and skin-protective properties, which make it a common ingredient in cosmetics [18]. However, QT has a tendency to lose its bioactivity in alkaline environments [19]. In addition, it is sensitive to temperature changes, with high temperatures making the hydroxyl groups of QT susceptible to oxidation [20], and limiting applications due to its low water solubility [21]. Research has shown that liposomes can increase the bioavailability and solubility of quercetin in the body. Priprem et al. [22] created liposomes containing quercetin with an encapsulation rate of nearly 80%. They combined egg yolk lecithin, cholesterol, and quercetin in a 2:1:1 ratio and dispersed them in a 50% polyethylene glycol water solution. Polymeric micelles also have the ability to greatly enhance the solubility of quercetin. Tan et al. [23] used a diblock copolymer called polyethylene glycol-derivatized phosphatidylethanolamine to produce nanomicelles loaded with quercetin using thin film dispersion. The encapsulation rate achieved was 88.9%, resulting in a remarkable 110-fold enhancement in the solubility of quercetin in water. Thus, combining the advantages of fatty acid vesicles and quercetin suggests that in cosmetic applications, fatty acid vesicles can not only enhance the stability of quercetin, but also expand its application.

In this study, we investigated the pH window migration and broadening effects of DA/TW80 composite vesicles at different molar ratios by mixing TW80 with DA and considering their stability. Polysorbate 80 (TW80) is a non-ionic surfactant with a long carbon chain structure that makes it easier to form inter and intramolecular interactions, such as hydrogen bonds and ionic bonds, and it has a good solubilizing effect on lipophilic drugs [24]. The polyethylene glycol chains of TW80 interact with water molecules in the aqueous phase, while its hydrophobic part collaborates with the hydrophobic portion of DA, enhancing the stability and prolonged effectiveness of the vesicles. This synergistic interaction enables these two molecules to jointly maintain the structure of the vesicle and control the drug release. QT was encapsulated in DA/TW80 composite vesicles to study the encapsulation rate, drug loading capacity, in vitro release behavior, skin permeability and retention properties, antioxidant activity, and antibacterial ability. The aim of this article is to evaluate the potential application value of DA/TW80 composite vesicles in the field of cosmetics.

## 2. Results and Discussion

### 2.1. Illustration of the Mechanisms for the Formation of Composite Solution System

**Figure 1:**
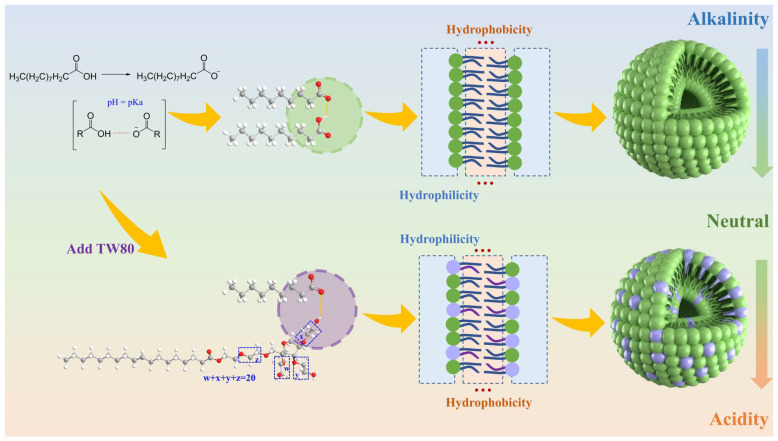
Schematic illustration of the formation of DA/TW80 composite vesicles.

The process of vesicle production involves complex physicochemical alterations [25]. Through an examination of the process of solution changes during DA vesicle formation, we have shown that the formation process can be broken down into several steps (Figure 1): (1) Under neutral or alkaline conditions, decanoic acid molecules are partially ionized to generate decanoic acid ions. (2) When the concentration of decanoic acid molecules reaches the critical micelle concentration (cmc), they will begin to aggregate spontaneously to form micelles. During this process, the decanoic acid molecules and the decanoic acid ions aggregate due to the hydrophobic effect, forming hydrogen bonds at the hydrophilic end. (3) The hydrophilic portions of the decanoic acid molecule and decanoic acid ion are close to each other, forming the outer layer of the membrane, while the hydrophobic portion is toward the inside of the membrane, resulting in a dual-layered membrane structure. (4) The continual aggregation of micelles and hydrogen bonding result in a large linear tension on the curved edge, causing both sides to contract inward and create a bilayer membrane vesicle structure.

Nevertheless, with the introduction of the surfactant TW80 to the DA, the concentration of decanoic acid ions required for the formation of the vesicle was reduced. The solubility of FAV in acidic conditions can be increased due to the specific solubilization of TW80 [26]. At the same time, the TW80 molecular chain consists of multiple polyethylene oxide structures and OH- groups, which can simultaneously form hydrogen bonds with various carboxylic acid groups nearby [27], partially replacing the original hydrogen bond between decanoic acid ions and decanoic acid. This facilitates the formation of vesicles, enabling the pH window of the DA/TW80 composite vesicles to migrate and expand.

### 2.2. Expansion of pH Window for DA/TW80 Composite Vesicles

**Figure 2:**
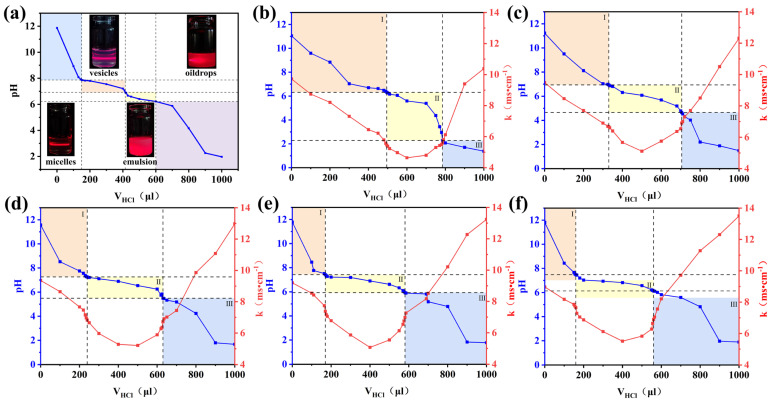
(**a**) The titration curve at room temperature for solution of decanoic acid. (**b**–**f**) The pH window of the DA/TW80 vesicles (R = 1:1; 1:2; 1:3; 1:4; 1:5).

We determined the pH window for the formation of DA vesicles by acid base titration [28]. As shown in Figure 2a, sodium decanoate forms a transparent micelle solution at a higher pH because of the repulsion between the carboxylate head groups in its surroundings [29]. With the addition of HCl, the pH was gradually decreases, and the carboxylate groups gradually protonates in the samples. When the amount of HCL reaches 150 μL, the concentration of DA reaches the critical vesicle concentration, and the solution starts to form vesicles with the blue opalescence phenomenon. When the HCL reaches 600 μL, the vesicle reaches saturation, and the surface tension begins to decline. At this point, the carboxylate ion gradually combines with H^+^ to form carboxylic acid molecules, which are insoluble in water, and the sample turns to milky white. This is followed by phase separation and water and oil separation. The titration curve shows the pH window of vesicle formation between 6.9 and 7.8 [4].

In order to expand the pH window for vesicle formation to meet the human skin, we mixed DA with TW80. The pH window of DA/TW80 composite vesicles was determined by combining acid base titration with the conductivity method [28]. A solution of DA (0.2 mol·L^−1^) was dissolved in NaOH solution (0.22 mol·L^−1^) and different proportions of TW80 were added to prepare the DA/TW80 sample solution, which was then titrated with 1 mol·L^−1^ HCL to achieve different pH values. The pH and conductivity were measured after a period of 3 days. Figure 2b–f show the titration curves of DA/TW80 composite vesicles at different molar ratios. Following the neutralization of excess NaOH with HCl, the pH titration curve appears to gradually decrease, and the conductivity decreases rapidly. When the NaOH is completely neutralized, HCl continues to react with sodium decanoate, and the carboxylate is gradually protonated. The pH decreases, and the conductivity decreases at a slower rate. With the continuous addition of HCl, sodium decanoate completely reacts, and the carboxylate is protonated to carboxylic acid. The excess HCl causes the pH to continue to decrease while the conductivity increases. When the vesicles form, the electrical conductivity reaches its minimum level. In the solution, surfactant or lipid molecules initially disperse as monomers. When the concentration reaches a certain critical point, the molecules spontaneously aggregate into micelles or vesicles. Changes in conductivity reflect the migration rate of ions in the solution, and the aggregation of surfactant or lipid molecules alters the ion concentration and the solvent properties of the solution. TW80 reduces the surface tension between the water and oil phases and promotes the dispersion and dissolution of decanoic acid molecules, facilitating the formation of vesicles with low pH conditions [26]. Table 1 lists the range and width of the pH window. According to the table, a higher value of R might cause a greater acid shift in the pH window. The pH window formed at R = 1:1 and 1:2 satisfy the pH range of human skin, and R = 1:2 was chosen for the subsequent experiments because less TW80 was required at R = 1:2, when the pH window was 4.65–6.94.

### 2.3. Characterization of DA/TW80 Composite Vesicles

**Figure 3:**
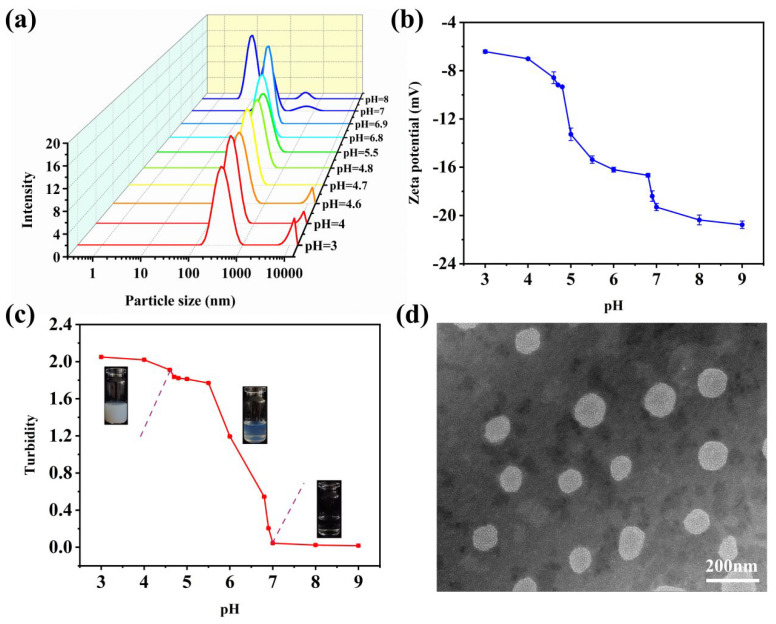
(**a**) Particle size, (**b**) zeta potential, (**c**) appearance pictures and turbidity of DA/TW80 composite solution system with different pHs (R = 1:2). (**d**) TEM of DA/TW80 vesicles at 200 nm (R = 1:2).

The self-assembly behavior of the DA/TW80 composite solution was investigated by dynamic light scattering (DLS). Figure 3a shows the particle size diagram of the DA/TW80 composite solution system with different pH values. Combined with the experimental results, it was inferred that the DA/TW80 composite solution experienced a process of aggregation formation followed by the aggregation disintegrating. When pH > 6.9, double peaks appear, and the presence of prominent peaks indicates the formation of aggregation, referred to as composite micelles. At this point, the particle size is relatively small, typically below 30 nm. Currently, when 4.7 < pH < 6.9, the DLS plot shows a single-peaked structure, resulting in the formation of composite vesicles consisting of DA/TW80. The average diameter of the vesicles, as determined by DLS, varies between 80 and 210 nm, and the particle size exhibits uniformity. At this time, the DA/TW80 composite vesicle system contains a higher quantity of carboxylate ions and carboxylate molecules, with both numbers equal. Additionally, pH < 4.7 produces two distinct peaks, and the second peak is sharp at this time. The small peak represents smaller particles in the aqueous phase or ions dissolved in the aqueous phase, while the large peak represents larger oil droplets or aggregates. This phenomenon is caused by the segregation of water and oil, as well as the disintegration of the vesicles in this system [30]. The particle distribution became polydispersion, indicating that the vesicle structure broke up due to electrostatic neutralization and the salt bridge [31].

To better understand the transformation process of the solution in the system, the zeta potential of the composite solution system was measured. The higher absolute value of zeta potential represents stronger repulsion among vesicles, thereby preventing aggregation of vesicles [32]. Therefore, zeta potential is considered a decent indicator of the interaction between aggregates. Figure 3b shows the variation in the zeta potential of the DA/TW80 solution at different pH values. It can be observed that the potential at all pH values exhibits negative potential values, and the absolute value of zeta potential increases as pH increases. When pH > 6.9, the dominant process is the deprotonation of DA to create micelles, resulting in a significant magnitude of the absolute value of zeta potential. When pH was at 4.7–6.9, a closed vesicle structure was formed in solution, leading to a significant amount of negative charge wrapping around the inner membrane of the vesicle [33]. The absolute value of zeta potential was found to be between 10 and 20 mV. As the pH decreases, the protonated DA forms an unstable emulsion, the vesicle structure disintegrates, and the absolute value of zeta potential is small.

Turbidimetry was utilized to investigate the changes in the aggregated phase of DA/TW80 composite vesicles at different pH values [34]. Figure 3c shows the turbidity change in DA/TW80 solution with different pH values at 400 nm. The absorbance of the composite solution decreases with the increase in pH. Alternatively, we observed that the self-assembly experienced a transition process of composite micellar to vesicle to emulsion [35]. When the pH exceeds 6.9, micelles appear in the solution, resulting in low turbidity and a transparent appearance. When the pH is between 4.7 and 6.9, there is a gradual increase in turbidity, and the solution exhibits a blue opalescent appearance. This indicates that the composite vesicles are formed at this time. At a pH below 4.7, the turbidity consistently increases, and the composite vesicles begin to disintegrate. This results in a turbid solution with a white appearance and the formation of an emulsion.

To obtain more information about the microstructural features of the composite vesicles, TEM was performed to characterize the morphology of the DA/TW80 vesicles in the samples at pH 5.5 (Figure 3d). It can be observed that a large number of vesicle structures were formed, and the vesicles were uniform in size and structure with unique spherical geometry. Because of the negative charge on the surface, the composite vesicles remained at a consistent distance from each other, and no adhesion occurred. The results confirm the formation of DA/TW80 vesicles [36].

### 2.4. FT-IR Analysis of DA/TW80 Composite Solution System

**Figure 4:**
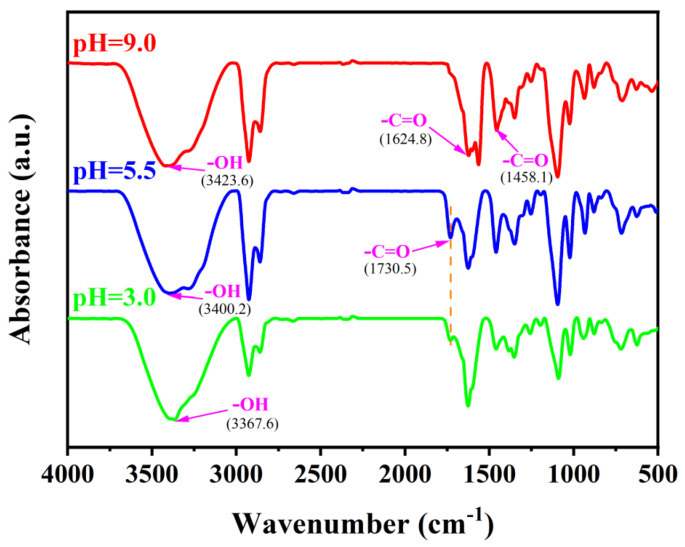
FT-IR of DA/TW80 composite solution (pH = 9.0, pH = 5.5, pH = 3.0).

FT-IR was utilized to investigate the inherent molecular interaction patterns in forming vesicles from DA/TW80 composite solution at different pH values (Figure 4) [37]. At pH 9.0, the peaks at 1624.8 cm^−1^ and 1458.1 cm^−1^ belong to the asymmetric and symmetrical stretching vibrations of the -C=O group in the carboxylic acid. The peak at 3423.6 cm^−1^ belongs to the stretching vibration of the -OH group in the carboxylic acid. When compared to pH 5.5, a new peak of -C=O appeared at 1730 cm^−1^, and the -OH peak at 3423.6 cm^−1^ was also moved to a low wavenumber and widened. This indicates that the effect of the -OH in the molecule is enhanced, which is a result of the formation of hydrogen bonds between the decanoic acid and the hydrophilic head group of the decanoic acid ion in the molecule [38]. With the continued decrease in pH, the -OH group moves to a low wavenumber of 3367.6 cm^−1^. The intensity of this peak decreased, and the intensity of the -C=O peak located in 1730.5 cm^−1^ also decreased, indicating that the intermolecular hydrogen bonding interaction gradually decreased with the decrease in pH. This is due to the conversion of carboxylate ions to carboxylate molecules, which causes a gradual rupture of the vesicular structure.

### 2.5. Stability of DA/TW80 Composite Vesicles

#### 2.5.1. Temperature Stability

From 10 °C to 60 °C, there was no significant alteration in particle size, and it remained in a steady state (Figure S1a). The absolute value of zeta potential exhibits a pattern of growing and then decreasing as the temperature rises (Figure S1b). It is possible that the temperature changed the surface charge of the DA/TW80 composite vesicles and influenced the magnitude of the zeta potential [39]. The turbidity of composite vesicles exhibited minimal variation across different temperatures (Figure S1c). The results indicate that the DA/TW80 composite vesicles remained relatively stable over a range of temperatures.

#### 2.5.2. Dilution Multiples Stability

As shown in Figure S2, the particle size remains relatively constant under different dilution multiples, with very modest variations in the absolute value of zeta potential. Additionally, the turbidity changes regularly during the dilution process, indicating the excellent stability of the composite vesicles upon dilution.

#### 2.5.3. Salt Stability

The particle size of the vesicles remained nearly constant as the concentration of NaCl increased, whereas the absolute value of the zeta potential gradually reduced (Figure S3a,b). The increase in salt concentration leads to an increase in ionic strength, causing compression of the double electric layer around the DA/TW80 composite vesicles and counterion binding between the cation and deprotonated composite vesicles [40]. The turbidity changes do not show any significant trend (Figure S3c), indicating that the DA/TW80 composite vesicles have good stability driven by hydrogen bonds.

#### 2.5.4. Storage Stability

**Figure 5:**
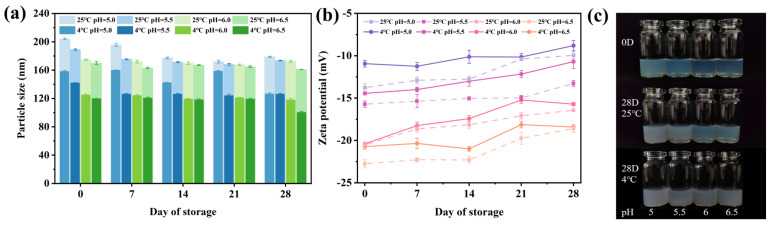
Variations in DA/TW80 composite vesicles during 28 days storage at 4 °C and 25 °C at pH = 5, 5.5, 6, 6.5. (**a**) Particle size, (**b**) zeta potential, (**c**) appearance picture.

The DA/TW80 composite vesicles were stored at 4 °C and 25 °C for 28 days to explore their storage stability at different temperatures. In Figure 5a, all vesicles showed no significant change in particle size after storing for 28 days, and all of the vesicles had particle sizes in the nanoscale range. During storage, it was discovered that the average vesicle diameter at 25 °C was larger than the average diameter at 4 °C. With the increase in storage time, the absolute value of the zeta potential of DA/TW80 composite vesicles decreased slightly, and the absolute value of the overall reduced zeta potential was around 5 mV (Figure 5b). According to Figure 5c, the turbidity increased slightly over time, but it still showed a blue opalescence. It was probably due to the small rupture and aggregation of vesicles that occurred during storage, but the structure of DA/TW80 composite vesicles was not destroyed. This indicates that the DA/TW80 composite vesicles have good storage stability.

### 2.6. FT-IR, DSC, and XRD Analysis of DA/TW80/QT Composite Vesicles

**Figure 6:**
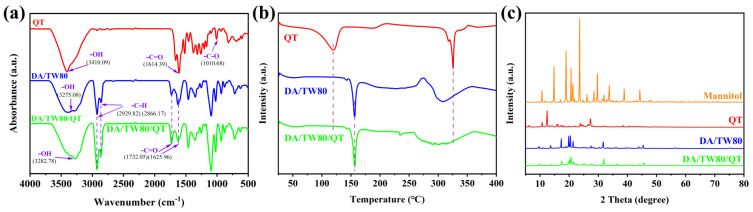
(**a**) FT-IR spectrogram, (**b**) DSC thermograms, (**c**) XRD of QT, DA/TW80, and DA/TW80/QT.

FT-IR, DSC, and XRD were measured for QT, DA/TW80 vesicles, and DA/TW80/QT composite vesicles, as illustrated in Figure 6. For quercetin, the peaks at 3410.1 cm^−1^, 1614.4 cm^−1^, and 1010.7 cm^−1^ correspond to the stretching vibrations of -OH, -C=O, and -C-O, respectively [41]. For the DA/TW80, -OH and -C=O peaks were formed at 2930.0–2860.0 cm^−1^ and 1735.0–1620.0 cm^−1^. The peaks corresponding to the -C=O bonds and -C-O bonds of quercetin were not detected in the DA/TW80/QT vesicles. Furthermore, the peaks in the blank vesicles were not significantly changed, suggesting that quercetin was successfully trapped within the vesicles (Figure 6a). To further confirm that QT was successfully encapsulated within the vesicles, we conducted DSC analysis on QT, DA/TW80 vesicles, and DA/TW80/QT composite vesicles. The results, as shown in Figure 6b, indicate distinct endothermic peaks at 120 °C and 325 °C for the QT sample. We observed an endothermic peak at 155 °C when testing DA/TW80. However, in the DA/TW80/QT sample, only the endothermic peak of DA/TW80 was observed, and the peaks at 120 °C and 325 °C, which are characteristic of QT, were not present. This confirms that QT was fully encapsulated within the DA/TW80 vesicles. In addition, The XRD analysis showed that when comparing the diffraction peaks of mannitol with those of other samples, the addition of mannitol during freeze-drying had almost no effect on the QT and decanoic acid vesicles. Distinct diffraction peaks corresponding to QT were clearly observed at 2θ = 10.7°, 11.6°, 14.7°, 23.6°, 26.0°, and 28.5° (Figure 6c) [42]. However, when examining the XRD pattern of the DA/TW80/QT vesicles, only peaks related to the blank vesicles were found, with no detection of QT. Furthermore, a comparison between the XRD images of DA/TW80 and DA/TW80/QT revealed that the positions of their diffraction peaks were nearly identical. This information collectively provides evidence that QT is encapsulated within the vesicles. All the data obtained from FT-IR, XRD, and DSC analysis revealed that the QT was successfully inserted into the vesicle bilayer, accompanied by the crystalline state changes and intermolecular interaction [43].

### 2.7. Encapsulation and In Vitro Release Behavior of DA/TW80/QT Composite Vesicles

**Figure 7:**
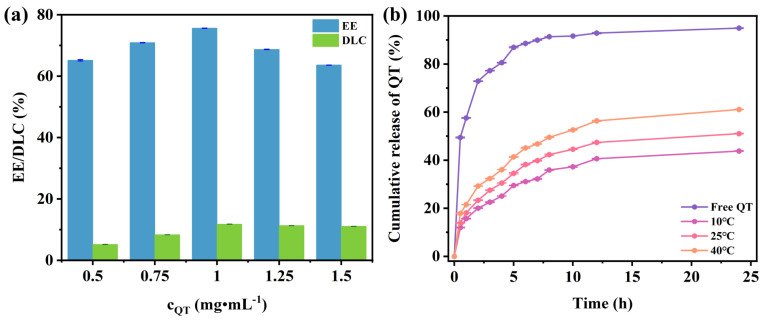
(**a**) EE and DLC at different QT concentrations (0.5–1.5 mg·mL^−1^). (**b**) The release curve of DA/TW80/QT composite vesicles at different temperatures (pH = 5.5).

The QT solution exhibits distinct UV absorption peaks at around 373 nm, and therefore, a UV spectrophotometer can be used to measure the EE and DLC of QT encapsulated by DA/TW80 composite vesicles. The absorbance of QT has a linear relationship with its concentration over a certain range, and the regression equation was y=0.06675x + 0.00955 (R^2^ = 0.99986) (Figure S4). QT is a lipid-soluble drug and is entrapped inside the vesicular bilayer. The encapsulated QT was applied to cosmetics in this study; therefore, pH = 5.5 was chosen to evaluate the EE and DLC at five different QT concentrations (0.5, 0.75, 1, 1.25, and 1.5 mg·mL^−1^). The choice of pH 5.5 is based on the average pH of human skin, which is 5.5 [44]. The EE and DLC results are presented in Figure 7a. When the QT concentration is very low, the probability of wrapping by composite vesicles is minimal, resulting in low EE and DLC. When the concentration of QT was increased to 0.5–1 mg·mL^−1^, both EE and DLC increased. With the continuous increase in QT concentration (1–1.5 mg·mL^−1^), the EE showed a decreasing trend, while DLC had a tendency to remain unchanged. The reason is that the ability of a definite concentration of composite vesicles to wrap the QT is limited, and the excess QT may cause the vesicle to become unstable and release the drug. Among these, when the concentration of QT was 1 mg·mL^−1^, DA/TW80 had the most capacity to wrap the QT. The EE was about 75.6%, and the DLC was 11.8%. At this time, the particle size of DA/TW80/QT composite vesicles was 191.98 ± 1.89 nm, and the absolute value of the zeta potential was 22.47 ± 1.18 mV (Figure S5). Therefore, the QT concentration of 1 mg·mL^−1^ was selected for subsequent studies.

Composite vesicles enable the sustained release of active ingredients, enhancing their applicability in cosmetics. Figure 7b shows the release curve of DA/TW80/QT vesicles at different temperatures, with free QT at 25 °C serving as a control. With the increasing temperature, the cumulative release rate of QT increases. The reason is perhaps that the increased temperature leads to enhanced thermal motion of the molecule, which promotes the release of QT from composite vesicles [34]. Free QT was released rapidly at 25 °C, while the release of QT encapsulated in DA/TW80 composite vesicles occurred at a slower rate. The cumulative release of QT at 10, 25, and 40 °C was 43.80%, 51.04%, and 61.10% at 24 h, respectively. These values are much lower than the 94.83% cumulative release in the unencapsulated control. DA/TW80 composite vesicles exhibit a significant ability to prolong the release of QT compared to free QT, indicating that DA/TW80 composite vesicles have a sustained release effect on QT. This demonstrates the potential application in a drug sustained delivery system.

Table 2 displays the equations and correlation coefficients (R^2^) obtained for DA/TW80/QT composite vesicles at different temperatures and free QT by fitting the four models. Comparison of the R^2^ values of the four models revealed that the R^2^ value of the Korsmeyer–Peppas model of free QT and DA/TW80/QT composite vesicles at different temperatures is closer to 1, suggesting that the kinetics fit the Korsmeyer–Peppas model.

### 2.8. In Vitro Skin Penetration Studies of DA/TW80/QT Composite Vesicles

**Figure 8:**
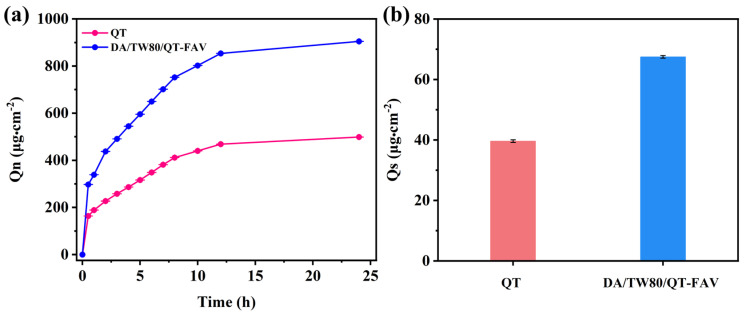
(**a**) Cumulative permeation, (**b**) skin retention of QT solution and DA/TW80/QT composite vesicles.

When employing a vesicular system in cosmetics, penetration analysis is an essential metric. The in vitro skin penetration behaviors of QT solution and DA/TW80/QT composite vesicles were investigated separately (Figure 8). Figure 8a shows that the release trajectories of DA/TW80/QT composite vesicles and QT solution are quite similar during the 0–24 h, and the cumulative penetration of the composite vesicles is more than that of the QT solution. The total amount of drug penetrated in 24 h from the QT solution was approximately 498.8 μg·cm^−2^, while the DA/TW80/QT composite vesicle was about 904.7 μg·cm^−2^, which is 1.81 times higher than that of the QT solution. This indicates that the skin permeability of DA/TW80/QT composite vesicles is superior to the QT solution. The reason is that the interaction between decanoic acid and the skin structure has a significant effect on increasing skin permeability. Thus, the DA/TW80 composite vesicles enhanced the skin permeability of QT solution to different extents. Furthermore, we observed that the DA/TW80/QT composite vesicles exhibited higher skin retention after 24 h and was 1.70 times more than that of the QT solution (Figure 8b). The high drug retention rate of composite vesicles may be due to the deposition of vesicles in the epidermis and forming the drug reservoir. Composite vesicles have excellent stability and high drug loading, allowing for efficient drug delivery to the deeper layers of the skin [45,46]. Therefore, using DA/TW80 composite vesicles as a drug carrier effectively extended the continuous transdermal time of the drug.

### 2.9. Antioxidant Capacity Studies of DA/TW80/QT Composite Vesicles

**Figure 9:**
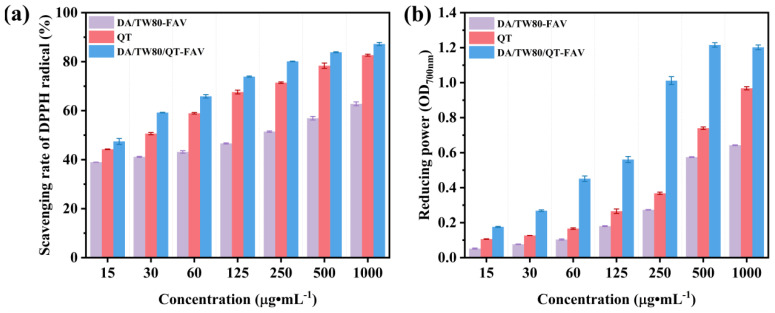
(**a**) DPPH radical scavenging activity, (**b**) FRAP of QT solution and DA/TW80/QT composite vesicles.

Composite vesicles allow the drug to better exert its antioxidant capacity, which is advantageous for cosmetic use. The antioxidant activity of DA/TW80/QT was evaluated using DPPH radical scavenging power and FRAP, with free QT serving as a control (Figure 9). Figure 9a shows the DPPH radical scavenging activity of free QT, DA/TW80 blank vesicles, and DA/TW80/QT composite vesicles at different concentrations. The DPPH radical scavenging ability of DA/TW80/QT composite vesicles, DA/TW80 blank vesicles, and QT increases progressively with increasing QT concentration. This is because a higher content of QT provides more hydrogen atoms to interact with DPPH radicals [41]. When we compared the scavenging ability of QT and blank vesicles, we found that the blank vesicles exhibited lower scavenging ability than QT. Compared to pure QT and blank vesicles, DA/TW80/QT composite vesicles exhibited higher activity, indicating that DA/TW80 vesicles work synergistically with QT to possess antioxidant activity. Additionally, we also investigated the ability of DA/TW80/QT composite vesicles to reduce Fe^3+^ to Fe^2+^ (Figure 9b). The higher the absorbance at 700 nm, the stronger the reducing ability. The results show that as the QT concentration increases, the reducing ability of DA/TW80/QT composite vesicles and QT also increases. Under different QT concentrations, the reduction in Fe^3+^ in DA/TW80/QT composite vesicles is always greater than that in QT and blank vesicles. This result aligns with the findings on the DPPH radical scavenging capacity. When QT is loaded into DA/TW80 composite vesicles, it effectively addresses the issue of poor water solubility. Therefore, the composite vesicles can effectively enhance the antioxidant capacity of QT, which is conducive to its application in cosmetic systems.

### 2.10. In Vitro Antibacterial Activity Studies of DA/TW80/QT Composite Vesicles

**Figure 10:**
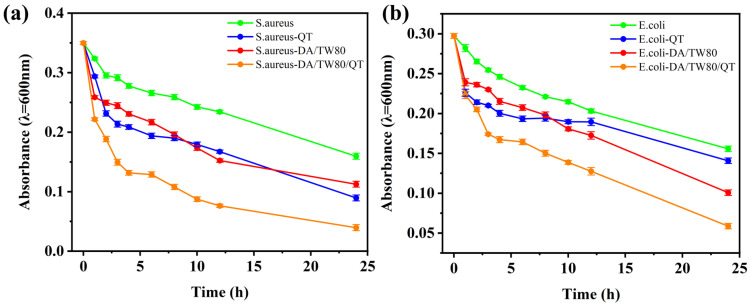
The antibacterial efficacy of QT, DA/TW80, DA/TW80/QT. (**a**) *S. aureus*, (**b**) *E. coli*.

The antibacterial effect of composite vesicles can reduce the dependence of cosmetics on traditional preservatives and improve the gentleness and safety of products. The antibacterial effects of QT, DA/TW80 vesicles, and DA/TW80/QT composite vesicles on *S. aureus* and *E. coli* were explored, respectively (Figure 10). The test was performed without oxygen, and the concentration of *S. aureus* and *E. coli* gradually decreased with time due to the bacteria being normally dead [47,48]. When QT is added, there will be a significant decrease in the quantities of *S. aureus* and *E. coli* initially, indicating that QT has an inhibitory effect on both bacteria. Both bacteria were similarly inhibited by the addition of blank vesicles. QT demonstrated greater efficacy in inhibiting *S. aureus*, while blank vesicles exhibited superior inhibition of *E. coli*. The QT was loaded into vesicles in order to investigate its bacteriostatic effect. Initially, it can be observed that DA/TW80/QT composite vesicles were significantly more effective than the other two, possibly owing to the interaction between the drug-loaded vesicles and the non-loaded QT. After 4 h, the *S. aureus* concentration exhibited a more gradual change, which can be attributed to the saturation of the inhibitory effect of drug-loaded vesicles on *S. aureus*. The concentration continued to decrease after 6 h due to the gradual release of the QT in the vesicles (Figure 10a). For *E. coli*, the concentration also exhibited a persistent reduction (Figure 10b). The results demonstrated that the inhibitory effect on the bacteria of DA/TW80/QT composite vesicles was persistent, and the inhibitory ability against *S. aureus* and *E. coli* was greater than that of drugs or vesicles alone.

## 3. Materials and Methods

### 3.1. Materials

Decanoic acid (DA), quercetin (QT), and potassium bromide (99.5% purity) were obtained from Maclin Biochemical Co., Ltd., Shanghai, China. Sodium hydroxide and hydrochloric acid were purchased from Sinopharm Chemical Reagent Co., Ltd., Shanghai, China. Polysorbate 80 was from Shanghai Een Chemical Technology Co., Ltd., Shanghai, China. Anhydrous ethanol and methanol were supplied by Tianjin Kaitong Chemical Reagent Factory, Tianjin, China. PBS phosphate buffer was provided by Sora Biotechnology Co., Beijing, China. The following chemicals were obtained from Shanghai Aladin Industries, Shanghai, China: 1,1-diphenyl-2-pyridyl hydrazine (DPPH), potassium ferrocyanide, trichloroacetic acid, and ferric chloride. *S. aureus* and *E. coli* were purchased from Bei Na Bio Ltd., Langfang, China. Triple-distilled water was used throughout the investigation.

### 3.2. Preparation of DA/TW80 Vesicles and DA/TW80/QT Vesicles

Weigh an appropriate amount of sodium hydroxide and add it to 100 mL of triple-distilled water to prepare a sodium hydroxide solution (0.22 mol·L^−1^). Then, weigh 3.45 g of DA and dissolve it in a small amount of anhydrous ethanol. Afterward, mix the decanoic acid solution with the sodium hydroxide solution to prepare a sodium decanoate solution. TW80 was dissolved in 0.1 mmol·L^−1^ PBS solution until it was completely dissolved. The molar ratio of TW80 to DA is 1:1 to 1:5 (R = nTW80: nDA). Sodium decanoate solution was mixed with TW80 solution and continuously stirred for 1 h at 1000 rpm in a magnetic stirrer. The compound solutions were subjected to pH adjustment with 1 mol·L^−1^ hydrochloric acid to produce a series of composite solutions with different pH. The temperature was consistently maintained at 40 °C during the entire process. The composite solution was allowed to remain at room temperature for 24 h. For the DA/TW80/QT vesicles, accurately weigh quercetin (5, 7.5, 10, 12.5, and 15 mg) and dissolve each amount in 10 mL of blank vesicle solution.

### 3.3. pH and Conductivity Measurements

The pH value of the composite solution was determined on a PHS-25 pH meter (Nanjing, China) with an E-201F glass electrode at 25 °C. Conductivity measurements were performed on a DDS-307A conductivity meter (Shanghai Yidian Scientific Instruments Co., Ltd., Shanghai, China) with a DJS-1C glass electrode at 25 °C. The conductivity of the distilled water was 3.0 μs·cm^−1^ at room temperature.

### 3.4. Dynamic Light Scattering (DLS) and Zeta Potential Measurements

The particle size of sample solutions was measured by the dynamic light scattering instrument (BT-90+, Dandong Bettersize Instrument Co. Ltd., Dandong, China). The zeta potential of sample solutions was measured using a Malvern Zetasizer Nano ZSE instrument (Malvern Instruments Ltd., Worcestershire, UK). Dynamic light scattering determines particle size by analyzing the diffusion of particles undergoing Brownian motion. DLS was used to measure the vesicle sizes in solution, employing a 633 nm He/Ne laser with the detector positioned at a 90° angle. Before measurement, we dilute all samples approximately 10 times with triply distilled water to prevent multiple scattering effects, reduce particle aggregation, and lower viscosity. Measurements were conducted at 25 °C, with an experimental equilibrium time of 120 s. We measured each sample three times and calculated the average size.

### 3.5. Turbidity Measurements

The turbidity of the composite solution was assessed using a UV-2450 ultraviolet spectrophotometer from Hitachi (Tokyo, Japan), employing quartz cells with a 1.0 cm path length. It measured at a wavelength of 400 nm. All turbidity measurements were conducted at room temperature.

### 3.6. Transmission Electron Microscopy (TEM)

The shape and surface morphology of decanoic acid vesicles were examined using a transmission electron microscope (TEM, Hitachi H-750, Tokyo, Japan). A sample was prepared by drop-casting onto carbon-coated copper grids. The surplus dispersion was eliminated using filter paper, and a 2.0 wt% phosphotungstic acid solution was added for complete staining. Then the liquid in excess was removed. The samples were dried and finally observed by transmission electron microscopy. An accelerating voltage of 120 kV was applied in a TEM analysis.

### 3.7. Fourier Transform Infrared Spectroscopy (FT-IR)

FT-IR was used to analyze the molecular interactions. Infrared measurements of the samples were carried out using Fourier transform infrared spectroscopy (FT-IR, VERTEX 70, Bruker Corporation, Karlsruhe, Germany). The freeze-dried solid samples were combined with potassium bromide in appropriate proportions, crushed, and pressed into thin films, and scanned within a full wave band in the range of 500–4000 cm^−1^.

### 3.8. Differential Scanning Calorimetry (DSC)

The phase transition temperature and chain melting temperature were measured using a differential scanning calorimeter (DSC, DTA-404PC, CSIMC, Beijing, China). Approximately 10 mg of the freeze-dried solid sample was weighed and placed in an aluminum pan, which was then sealed. The measuring range of temperature was from 20 to 400 °C at a rate of 10 °C·min^−1^. Empty pans were used as references.

### 3.9. X-Ray Diffraction (XRD)

The crystal structure of the samples was determined by X-ray diffraction (XRD, Rigaku Ultima IV, Tokyo, Japan). The scanning speed was 10°·min^−1^ and the scanning range was 5–80°, using a Cu Kα source (λ = 1.5406 Å) and radiating at 40 kV and 50 mA in air.

### 3.10. Stability Studies

Composite vesicle stability plays an important role in cosmetic applications. Consequently, the stability of the DA/TW80 composite vesicle was assessed under various conditions, including temperature, dilution multiples, salt concentration, and storage time. Each sample was measured three times.

#### 3.10.1. Temperature Stability

Temperature significantly influences the self-assembled structure of fatty acids due to the chain melting process [49]. “Chain melting” refers to the transition of hydrocarbon chains in lipids from an organized crystalline state to a disordered liquid crystal state [50]. The DA/TW80 composite vesicles were subjected to various temperatures (10, 20, 30, 40, 50, and 60 °C) by immersion in a thermostatic water bath. Furthermore, we assessed particle size, zeta potential, and turbidity after equilibrating each sample at varying temperatures for 30 min.

#### 3.10.2. Dilution Multiples Stability

In the actual application process, DA/TW80 is added to the products and generally needs to be diluted before application, which requires the vesicles to have excellent dilution stability. To examine the impact of various dilution multiples on the stability of the system, composite vesicles were diluted at multiples of 10, 20, 40, 60, 80, and 100 using triple-distilled water and the particle size, zeta potential, and turbidity were measured.

#### 3.10.3. Salt Stability

Fatty acids are an important component of biological cells, and the fusion of fatty acid vesicles is associated with the fusion of membranes in real biological cells. Membrane fusion occurs in an electrolyte environment in biological cells [51]. Therefore, we investigated the stability of the composite vesicles in an electrolyte environment. We prepared sodium chloride solutions with different salt concentrations (0, 20, 40, 60, 80, and 100 mM). Then, the DA/TW80 composite vesicles were combined with different concentrations of sodium chloride solution at a 1:5 volume ratio and kept at room temperature for 12 h until measurement.

#### 3.10.4. Storage Stability

The composite vesicles were stored at 4 °C and 25 °C for a duration of 28 days in order to assess their stability. We measured their particle size and zeta potential at fixed intervals (0, 7, 14, 21, and 28 days). The composite vesicles were placed in a transparent glass bottle tightly covered with aluminum foil to assess their stability.

### 3.11. Encapsulation Efficiency and Drug Loading Capacity of DA/TW80/QT Vesicles

The encapsulation efficiency (EE) of the vesicles is described as the ratio of the amounts of drugs that are encapsulated in the vesicle relative to the total amount of drugs employed in the composite vesicle. Drug loading capacity (DLC) is determined by the ratio of the number of encapsulated drugs to the total amount of vesicles [9]. Quercetin (QT) was selected as the model drug to evaluate the encapsulation efficiency and drug loading capacity of the samples. Precisely 1 mL of sample was added to a 10 mL volumetric flask, followed by methanol, and the sample was shaken well and sonicated for 10 min in order to release QT. The total concentration was calculated as C_1_ at a wavelength of 373 nm using an ultraviolet spectrophotometer (UV-2450, Hitachi, Tokyo, Japan), and the corresponding solute mass was calculated as W_1_. After centrifugation for 0.5 h at 10,000 rpm, the resultant supernatant was fixed with methanol to 10 mL, the free QT concentration was calculated as C_2_, and the corresponding solute mass was calculated as W_2_. The quality of the freeze-dried powder of DA/TW80/QT was denoted as W_3_, and the following Formulas (1) and (2) were used to calculate the encapsulation efficiency (EE) and drug loading capacity (DLC) [52]:

(1) $$EE(\%)=\frac{W_{1}-W_{2}}{W_{1}}\times 100\%$$

(2) $$DLC(\%)=\frac{W_{1}-W_{2}}{W_{3}}\times 100\%$$

where W_1_ is the weight of QT added, W_2_ is the amount of free QT, and W_3_ is the weight of the mixture (DA, TW80, and loaded encapsulated QT).

### 3.12. In Vitro Drug Release Studies

The dialysis approach was used to analyze the release behavior of QT from DA/TW80/QT vesicles. Dialysis bags (cutoff 8000–14,000 Da) containing 4 mL of DA/TW80/QT vesicles were immersed in 200 mL of PBS solution containing 0.5% (*v*/*v*) TW80 with a pH of 5.5 and stirred at 10, 25, and 40 °C. A magnetic stirrer was employed to slowly stir the release medium. The release medium (3 mL) was removed at the 0.5, 1, 2, 3, 4, 5, 6, 8, 10, 12, 24 h time points and replaced with the same volume of fresh phosphate buffer. The concentration of withdrawn samples was determined by a UV spectrophotometer. In addition, the study also examined the release of pure QT for comparison. The cumulative release rate was then calculated according to the following equation [53,54]:

(3) $$Cumulative\;release\;of\;drug=\frac{c_{n}\times V_{0}+\sum_{i=1}^{n-1}c_{i}\times V_{i}}{m}\times 100\%$$

where c_n_ is the concentration of QT in the release fluid at the nth sample time point (μg·mL^−1^), V_0_ is the total volume of the release medium (mL), c_i_ is the concentration of QT in the release fluid at the i (I = n − 1) sample time point (μg·mL^−1^), V_i_ is the volume of the release medium removed at each time (mL), and m is the total mass of QT in the vesicles in the dialysis bag (mg).

Kinetic modeling of the Zero order, First order, Higuchi, and Korsmeyer–Peppas release equations was applied to explain the release mechanism of QT in Table 3. By comparing the regression coefficients R2, the model with an R2 value closer to 1 was considered the best-fitting model [55].

### 3.13. In Vitro Skin Penetration and Skin Retention Test

The measurement of the skin permeability (Q_n_) and skin retention (Q_m_) of the DA/TW80/QT composite vesicles was conducted using a YB-P6 Franz diffusion cell (Tianjin Pharmacopoeia Standard Instrument Factory, Tianjin, China). The mouse skin diffusion membrane was positioned between the donor and receptor chambers, with the stratum corneum facing the donor chamber. The surface area available for diffusion was 1.766 cm^2^. The receptor chamber was constructed by introducing 15 mL of a PBS solution containing 0.5% (*v*/*v*) TW80 (pH 6.8). Then, 2 mL of the DA/TW80/QT composite vesicles and 2 mL of the QT solution were introduced into the donor chamber, respectively. The chamber was then sealed tightly with parafilm. The temperature of the receptor media was sustained at 37 ± 0.5 °C and was constantly agitated at a rate of 300 rpm for the whole length of the experiment. At different time intervals, 2 mL samples were collected from the receiving fluid and then replaced with an equivalent volume of fresh receiver fluid. The drug concentration in the receiving solution was analyzed using a UV spectrophotometer. The cumulative transmittance (Q_n_) of skin per unit area at each time point was calculated using the formula [56], which is as follows:

(4) $$Q_{n}=\frac{V_{0}\times C_{n}+\sum_{i=1}^{n-1}\left(C_{i}\times V_{i}\right)}{A}$$

where *V*_0_ is the total volume of the releasing medium (mL), *C*_n_ is the drug concentration at the nth point (μg·mL^−1^); *C*_i_ is the drug concentration at the i (I = n − 1) point (μg·mL^−1^); *V*_i_ is the volume of the sample solution at time i (mL); and *A* is the effective diffusion area of the Franz diffusion cell (cm^2^).

The amount of QT that remained in the skin was determined after the in vitro permeation experiment was concluded (24 h). The skin surface was cleaned with normal saline and then wiped with filter paper to remove the residual drug. Subsequently, the cleaned skin was sliced into small fragments, and the tissue was further homogenized with 20 mL of ethanol. The resultant mixture was then kept at room temperature for a duration of 6 h to ensure thorough extraction of the drug. The quantity of QT retained on the skin was analyzed using a UV spectrophotometer. The calculation of intradermal retention per unit area (Q_m_) was established according to the equation [56], as follows: *C*_m_ is the extract concentration (μg·mL^−1^), *V* is the extract volume (mL), and *A* is the percutaneous penetration area (cm^2^). Every investigation was conducted three times.

(5) $$Q_{m}=\frac{C_{m}\times V}{A}$$

### 3.14. Antioxidant Activity Studies

The DPPH radical scavenging activity was assayed according to the literature with slight modifications [57]. Samples were produced at various concentrations (15.625–1000 µg·mL^−1^) from the stock solution (1 mg·mL^−1^). The DPPH compound was dissolved in ethanol to create a 0.20 mmol·L^−1^ DPPH ethanol solution. Subsequently, a 100 µL volume of DPPH solution was combined with an equivalent volume of the samples. The mixture was incubated for 30 min at 25 °C in the dark. The absorbance of the samples at 517 nm was measured using an enzyme-linked immunosorbent assay (Thermo Fisher Scientific Corporation, Vantaa, Finland). Triple-distilled water combined with the DPPH solution served as the control, whereas ethanol was utilized in place of the DPPH solution as the blank control. The experiment was repeated three times. The DPPH radical scavenging activity was calculated using the following equation:

(6) $$DPPH\;scavenging\;activity\;(\%)=\frac{1-(A_{s}-A_{i})}{A_{0}}$$

where *A*_s_ is the absorbance of the sample at 517 nm, *A*_i_ is the absorbance of the blank, and *A*_0_ is the absorbance of the control sample.

The ferric reducing antioxidant power (FRAP) was evaluated in accordance with the literature, with minor adjustments [58]. Of the sample solution, 1 mL was mixed with 1 mL of phosphate buffer (pH 6.6, 0.2 mol·L^−1^) and 1 mL of 1% potassium ferricyanide solution. The mixture was incubated for 20 min at 50 °C, then 1 mL of 10% trichloroacetic acid was introduced, and the resultant mixture was centrifuged at 5000 rpm for 10 min. Next, 1 mL of the supernatant was combined with 1 mL of triple-distilled water and 0.2 mL of 0.1% ferric chloride solution. The samples were thereafter maintained in a dark atmosphere for 10 min, after which their absorbance was measured at 700 nm. A solution without ferric chloride was used as a control.

### 3.15. In Vitro Antibacterial Ability Study

In order to investigate the antibacterial ability of DA/TW80/QT complex vesicles, we conducted separate tests using *Staphylococcus aureus* (*S. aureus*) and *Escherichia coli* (*E. coli*) [59,60]. The purified bacteria were incubated in a liquid medium in a 37 °C constant temperature shaker for 12 h. DA/TW80 vesicles, DA/TW80/QT composite vesicles, and QT solutions of equal concentration were prepared, and 100 µL of solution was dissolved in 1 mL of *S. aureus* or *E. coli*. Then, the samples were incubated for 0, 1, 2, 3, 4, 6, 8, 10, 12, and 24 h, and the absorbance was measured using a UV spectrophotometer. The inhibition of QT, blank vesicles, and drug-loaded vesicles on bacteria was observed.

## 4. Conclusions

In this study, the addition of the non-ionic surfactant TW80 can migrate and extend the pH window of DA-forming vesicles. The results indicate that the formation of DA/TW80 composite vesicles is primarily driven by hydrogen bonding, and the pH windows of vesicle formation can be regulated from the original 6.90–7.80 to 2.28–6.31. Hence, vesicles can exist within the pH range adapted to the human living system. These composite vesicles exhibited excellent stability across various conditions, including changes in temperature, dilution, salt concentration, and prolonged storage. The in vitro release behavior demonstrated that DA/TW80/QT composite vesicles provided a delayed release compared to free QT, indicating their sustained-release capability. Furthermore, in vitro percutaneous penetration studies demonstrated that the combination of DA/TW80/QT composite vesicles exhibited superior skin permeability and retention properties. These results suggested using composite vesicles as a cosmetics carrier effectively extended the continuous transdermal time of the drug. Antioxidant activity assays revealed that the DA/TW80/QT composite vesicles exhibited greater DPPH radical scavenging and iron ion reduction capacities than the QT solution. The antibacterial studies showed that the inhibitory impact of composite vesicles on *S. aureus* and *E. coli* increased over time and was significantly greater than that of QT. Therefore, the composite vesicles can effectively enhance the antioxidant capacity and antibacterial activity of QT, which is conducive to its application in cosmetic systems. The above results suggest that DA/TW80 composite vesicles have great potential in the cosmetic industry, and further studies will focus on the topical application of composite vesicles as cosmetics carriers.

## Figures and Tables

**Table 1:** The effect of R on the DA/TW80 vesicles pH window (R = 1:1–1:5).

R	pH Window	
1:1	2.28–6.31	4.03
1:2	4.65–6.94	2.29
1:3	5.49–7.26	1.77
1:4	5.93–7.42	1.49
1:5	6.13–7.48	1.35

**Table 2:** Fits of four kinds of release kinetic models for QT at different temperatures.

Temperature (°C)	Mode	Equation	R^2^
10	Zero order	F = 0.0158t + 0.1655	0.6780
	First order	−ln(1 − F) = 0.0221t + 0.1821	0.7443
	Higuchi	F = 0.0931t^0.5^ + 0.0617	0.9270
	Korsmeyer–Peppas	lgF = 0.3615lgt − 0.8024	0.9820
25	Zero order	F = 0.0185t + 0.1991	0.6547
	First order	−ln(1 − F) = 0.0277t + 0.2249	0.7348
	Higuchi	F = 0.1097t^0.5^ + 0.0754	0.9158
	Korsmeyer–Peppas	lgF = 0.3679lgt − 0.7321	0.9780
40	Zero order	F = 0.0218t + 0.2385	0.6630
	First order	−ln(1 − F) = 0.0367t + 0.2738	0.7729
	Higuchi	F = 0.129t^0.5^ + 0.0937	0.9202
	Korsmeyer–Peppas	lgF = 0.351lgt − 0.6428	0.9808
25 (QT)	Zero order	F = 0.0247t + 0.5925	0.3623
	First order	−ln(1 − F) = 0.1112t + 1.0654	0.6750
	Higuchi	F = 0.1697t^0.5^ + 0.3768	0.6772
	Korsmeyer–Peppas	lgF = 0.1814lgt − 0.2157	0.9094

**Table 3:** Original and rewritten formula of the drug-releasing kinetic models.

Kinetic Models	Original Formula	Rewritten Formula [55]
Zero order	$F=k_{0}t$	$F=k_{0}t$
First order	$F=1-e^{-k_{1}t}$	$-ln(1-F)=k_{1}t$
Higuchi	$F=k_{H}t^{0.5}$	$F=k_{H}t^{0.5}$
Korsmeyer–Peppas	$F=k_{kp}t^{n}$	$lgF=nlgt+lgk_{kp}$

## Data Availability

The data that support the findings of this study are available from the corresponding author upon reasonable request.

## Supplementary Materials

The following supporting information can be downloaded at: https://www.mdpi.com/article/10.3390/molecules30030624/s1, Figure S1: Variations of DA/TW80 composite vesicles with different temperature at pH = 5, 5.5, 6, 6.5; Figure S2: Variations of DA/TW80 composite vesicles for different dilution multiples at pH = 5, 5.5, 6, 6.5; Figure S3: Variations of DA/TW80 composite vesicles in NaCl solution with different concentrations at pH = 5, 5.5, 6, 6.5; Figure S4: Ultraviolet spectrogram and Standard curve of QT; Figure S5: Particle size and Zeta potential of DA/TW80/QT composite vesicles.
