# Supplementary material for: Design of Decanoic Acid/Polysorbate 80 Composite Vesicles as Cosmetics Carrier: Stability, Skin Permeability, Antioxidant and Antibacterial Activity
Source: Molecules. 2025 Jan 31;30(3):624. doi: 10.3390/molecules30030624 (PMC11821021; doi:10.3390/molecules30030624)
Supplement: Supplementary file 1 [file molecules-30-00624-s001.zip › molecules-3428480-supplementary.pdf]

Supporting Information

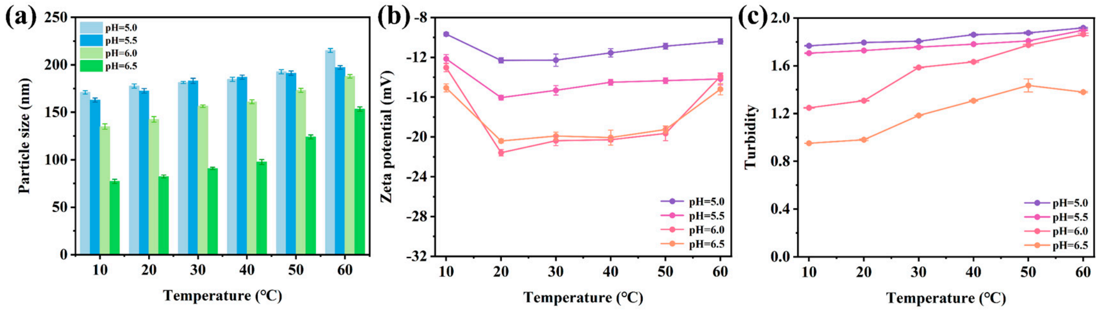

Figure S1 Variations of DA/TW80 composite vesicles with different temperature (10-60 ℃) at pH= 5, 5.5, 6, 6.5. (a) Particle size (b) Zeta potential (c) Turbidity.

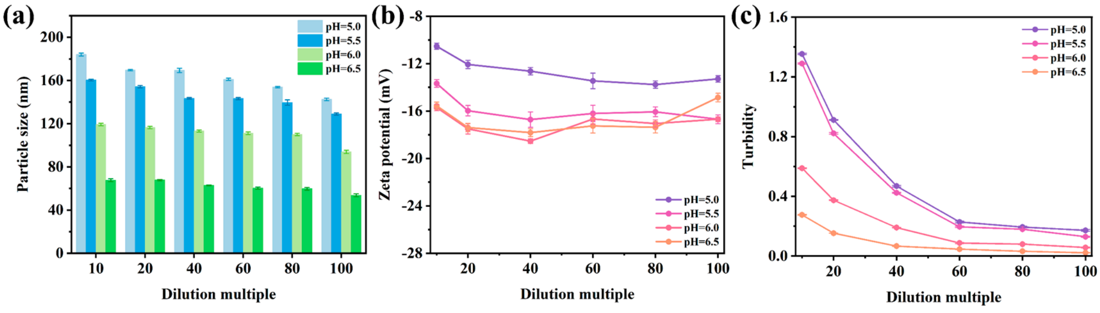

Figure S2 Variations of DA/TW80 composite vesicles for different dilution multiples (10-100) at pH= 5, 5.5, 6, 6.5. (a) Particle size (b) Zeta potential (c) Turbidity.

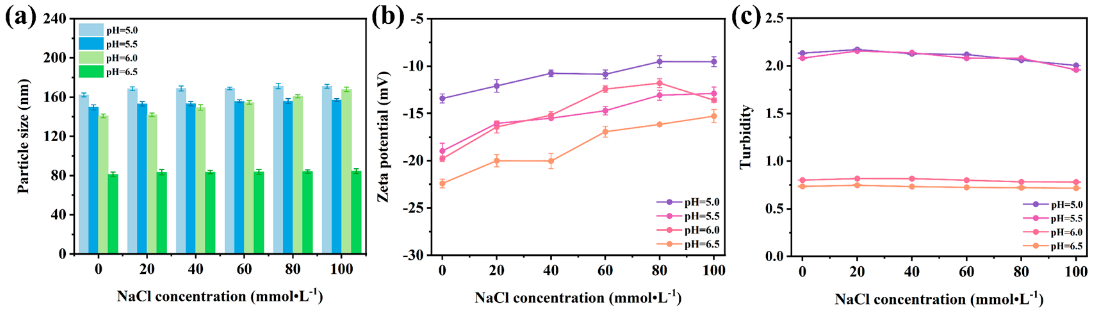

Figure S3 Variations of DA/TW80 composite vesicles in NaCl solution with different concentrations (0-100 mM) at pH= 5, 5.5, 6, 6.5. (a) Particle size (b) Zeta potential (c) Turbidity.

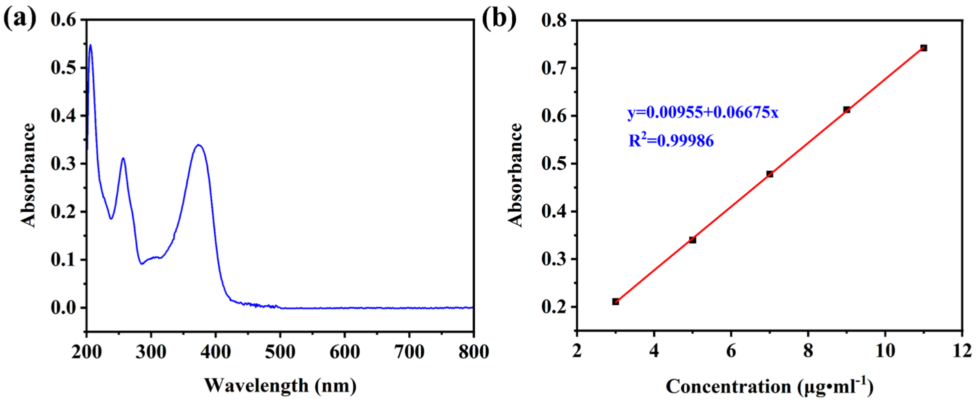

Figure S4 (a) Ultraviolet spectrogram (b) Standard curve of QT.

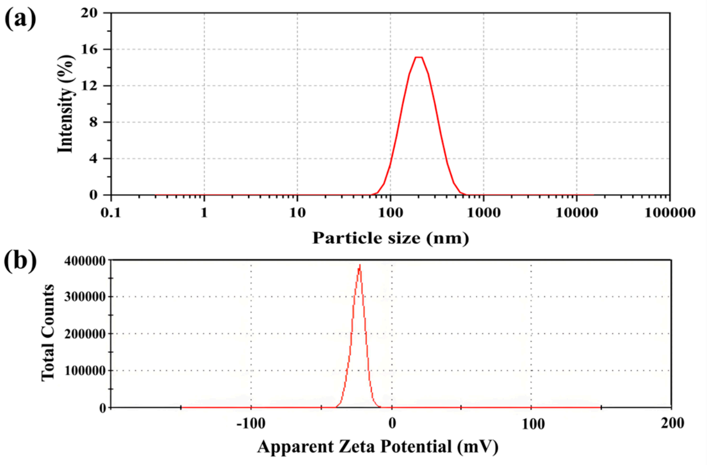

Figure S5 (a) Particle size (b) Zeta potential of DA/TW80/QT composite vesicles (c_{QT}=1 mg·mL^{-1}).
